# Supplementary material for: Design and Immunoinformatic Assessment of Candidate Multivariant mRNA Vaccine Construct against Immune Escape Variants of SARS-CoV-2
Source: Polymers (Basel). 2022 Aug 10;14(16):3263. doi: 10.3390/polym14163263 (PMC9414445; doi:10.3390/polym14163263)
Supplement: Supplementary file 1 [file polymers-14-03263-s001.zip › File S6.pdf]

(a) DNA Sequence of DOW-21 for *in vitro* transcription

GGC AAA AAT CAA AAT CAA TCA TCA TCA CAA CAT CAA CAA TCA ATC ATC AAC
ACA TCA TCAAGA CAC CAC C ATGGAAACCC CGGCGCAGCT GCTGTTTCTG
CTGCTGCTGT GGCTGCCGGATACCACCGGCAGC CAG TGC GTG AAC CTG CGC ACC
AGG ACG CAG CTG CCG CCG GCC TAC ACC AAC AGC TTC ACC CGG GGC GTG TAC
TAC CCC GAC AAG GTG TTC CGC TCC AGC GTG CTG CAC TCC ACC CAG GAC CTG
TTC CTG CCG TTC TTC AGC AAC GTG ACC TGG TTC CAC GCC ATC TCC GGC ACC
AAC GGC ACC AAG CGG TTC GAC AAC CCC GTG CTG CCC TTC AAC GAC GGG GTG
TAC TTC GCC AGC ACC GAG AAG TCC AAC ATC ATC CGC GGC TGG ATC TTC GGC
ACC ACC CTG GAC AGC AAG ACC CAG AGC CTG CTG ATC GTG AAC AAC GCC ACC
AACGTG GTC ATC AAG GTG TGC GAG TTC CAG TTC TGC AAC GAC CCC TTC CTG
GAC CAC AAG AAC AAC AAG AGC TGG ATG GAG AGC GGC GTG TAC AGC AGC GCC
AAC AAC TGC ACC TTC GAG TAC GTG TCC CAG CCG TTC CTG ATG GAC CTG GAG
GGC AAG CAG GGC AAC TTC AAG AAC CTG CGC GAG TTC GTG TTC AAG AAC ATC
GAC GGC TAC TTC AAG ATC TAC AGC AAG CAC ACC CCG ATC ATC GTG CGG GAG
CCC GAG GAC CTG CCG CAG GGC TTC TCG GCG CTG GAG CCC CTG GTG GAC CTG
CCC ATC GGC ATC AAC ATC ACC CGG TTC CAG ACG CTG CAC CGC AGC TAC CTG
ACC CCC GGC GAC AGC AGC AGC GGG TGG ACG GCG GGC GCC GCC GCG TAC TAC
GTG GGC TAC CTG CAG CCG CGG ACC TTC CTG CTG AAG TAC AAC GAG AAC GGC
ACC ATC ACC GAC GCC GTG GAC TGC GCG CTG GAC CCG CTG AGC GAG ACG AAG
TGC ACC CTG AAGGGA GGA GGG GCG GGA GGG GCA GGA GGG GCGCGG GTG CAG
CCC ACC GAG TCC ATC GTG CGG TTC CCC AAC ATC ACC AAC CTG TGC CCC TTC
GGC GAG GTG TTC AAC GCC ACC CGC TTC GCC TCC GTG TAC GCC TGG AAC CGG
AAG CGG ATC AGC AAC TGC GTG GCC GAC TAC TCC GTG CTG TAC AAC TCC GCC
AGC TTC AGC ACC TTC AAG TGC TAC GGC GTG TCC CCG ACC AAG CTG AAC GAC
CTG TGC TTC ACG AAC GTG TAC GCC GAC AGC TTC GTG ATC CGG GGG GAC GAG
GTG CGG CAG ATC GCC CCC GGC CAG ACG GGC AAC ATC GCC GAC TAC AAC TAC
AAG CTG CCC GAC GAC TTC ACC GGC TGCGTG ATC GCC TGG AAC AGC AAC AAC
CTG GAC TCC AAG GTC GGC GGC AAC TAC AAC TAC CGC TAC CGG CTG TTC CGG
AAG TCC AAC CTG AAG CCC TTC GAG CGG GAC ATC TCC ACC GAG ATC TAC CAG
GCC GGC AAC AAG CCG TGC AAC GGC GTG AAG GGC TTC AAC TGC TAC TTC CCG
CTG CAG TCC TAC GGC TTC CAG CCC ACG TAC GGC GTG GGC TAC CAG CCC TAC
AGG GTG GTG GTG CTG AGC TTC GAG CTG CTG CAC GCC CCG GCC ACC GTG TGC
GGC CCG AAG AAG AGC ACC AAC CTC GTG AAG AAC AAG TGC GTG AAC
TTCTAATTGTGTATGCGTTAATAAAAAGAAGGAACTCGTAAAAACTCAATGTATTTC
TGAGGAAGCGTGGTGCATAATGCCACGCAGCGTCTGCATAACTTTTATTATTTCTTTT
ATTAATCAACAAAAAAAAAAAAAAAAAAAAAAAAAAAAAAAAAAAAAAAAAAAA
AAAAAAAAAAAAAAAAAAAAAAAAAAAAAAAAAAAAAAAAAAAAAAAAAAAAAA
AAAAAAAAAAAAAAAAAAAAAAAAA

(b) *In vitro* transcribed sequence of DOW-21 (Unmodified)

GGC AAA AAU CAA AAU CAA UCA UCA UCA CAA CAU CAA CAA UCA AUC AUC AAC ACA UCA UCAAGA CAC CAC C AUGGAAACCC CGGCGCAGCU GCUGUUUCUG CUGCUGCUGU GGCUGCCGGAUACCACCGGCAGC CAG UGC GUG AAC CUG CGC ACC AGG ACG CAG CUG CCG CCG GCC UAC ACC AAC AGC UUC ACC CGG GGC GUG UAC UAC CCC GAC AAG GUG UUC CGC UCC AGC GUG CUG CAC UCC ACC CAG GAC CUG UUC CUG CCG UUC UUC AGC AAC GUG ACC UGG UUC CAC GCC AUC UCC GGC ACC AAC GGC ACC AAG CGG UUC GAC AAC CCC GUG CUG CCC UUC AAC GAC GGG GUG UAC UUC GCC AGC ACC GAG AAG UCC AAC AUC AUC CGC GGC UGG AUC UUC GGC ACC ACC CUG GAC AGC AAG ACC CAG AGC CUG CUG AUC GUG AAC AAC GCC ACC AACGUG GUC AUC AAG GUG UGC GAG UUC CAG UUC UGC AAC GAC CCC UUC CUG GAC CAC AAG AAC AAC AAG AGC UGG AUG GAG AGC GGC GUG UAC AGC AGC GCC AAC AAC UGC ACC UUC GAG UAC GUG UCC CAG CCG UUC CUG AUG GAC CUG GAG GGC AAG CAG GGC AAC UUC AAG AAC CUG CGC GAG UUC GUG UUC AAG AAC AUC GAC GGC UAC UUC AAG AUC UAC AGC AAG CAC ACC CCG AUC AUC GUG CGG GAG CCC GAG GAC CUG CCG CAG GGC UUC UCG GCG CUG GAG CCC CUG GUG GAC CUG CCC AUC GGC AUC AAC AUC ACC CGG UUC CAG ACG CUG CAC CGC AGC UAC CUG ACC CCC GGC GAC AGC AGC AGC GGG UGG ACG GCG GGC GCC GCC GCG UAC UAC GUG GGC UAC CUG CAG CCG CGG ACC UUC CUG CUG AAG UAC AAC GAG AAC GGC ACC AUC ACC GAC GCC GUG GAC UGC GCG CUG GAC CCG CUG AGC GAG ACG AAG UGC ACC CUG AAGGGA GGA GGG GCG GGA GGG GCA GGA GGG GCGCGG GUG CAG CCC ACC GAG UCC AUC GUG CGG UUC CCC AAC AUC ACC AAC CUG UGC CCC UUC GGC GAG GUG UUC AAC GCC ACC CGC UUC GCC UCC GUG UAC GCC UGG AAC CGG AAG CGG AUC AGC AAC UGC GUG GCC GAC UAC UCC GUG CUG UAC AAC UCC GCC AGC UUC AGC ACC UUC AAG UGC UAC GGC GUG UCC CCG ACC AAG CUG AAC GAC CUG UGC UUC ACG AAC GUG UAC GCC GAC AGC UUC GUG AUC CGG GGG GAC GAG GUG CGG CAG AUC GCC CCC GGC CAG ACG GGC AAC AUC GCC GAC UAC AAC UAC AAG CUG CCC GAC GAC UUC ACC GGC UGCGUG AUC GCC UGG AAC AGC AAC AAC CUG GAC UCC AAG GUC GGC GGC AAC UAC AAC UAC CGC UAC CGG CUG UUC CGG AAG UCC AAC CUG AAG CCC UUC GAG CGG GAC AUC UCC ACC GAG AUC UAC CAG GCC GGC AAC AAG CCG UGC AAC GGC GUG AAG GGC UUC AAC UGC UAC UUC CCG CUG CAG UCC UAC GGC UUC CAG CCC ACG UAC GGC GUG GGC UAC CAG CCC UAC AGG GUG GUG GUG CUG AGC UUC GAG CUG CUG CAC GCC CCG GCC ACC GUG UGC GGC CCG AAG AAG AGC ACC AAC CUC GUG AAG AAC AAG UGC GUG AACUUCUAAUUGUGUAUGCGUUAAUAAAAGAAGGAACUCGUAAAAACUCAAUG UAUUUCUGAGGAAGCGUGGUGCAUAAUGCCACGCAGCGUCUGCAUAACUUUUAU UAUUUCUUUUAUUAAUCAACAAAAAAAAAAAAAAAAAAAAAAAAAAAAAAAAAAAA AAAAAAAAAAAAAAAAAAAAAAAAAAAAAAAAAAAAAAAAAAAAAAAAAAAAAAAA AAAAAAAAAAAAAAAAAAAAAAAAAAAAAAAAAAAA

(c) *In vitro* transcribed sequence of DOW-21 (Modified Uracil)

GGC AAA AAΨ CAA AAΨ CAA ΨCA ΨCA ΨCA CAA CAΨ CAA CAA ΨCA AΨC AΨC AAC ACA ΨCA ΨCAAGA CAC CAC C AΨGGAAACCC CGGCGCAGCΨ GCΨGΨΨΨCΨG CΨGCΨGCΨGΨ GGCΨGCCGGAΨACCACCGGCAGC CAG ΨGC GΨG AAC CΨG CGC ACC AGG ACG CAG CΨG CCG CCG GCC ΨAC ACC AAC AGC ΨΨC ACC CGG GGC GΨG ΨAC ΨAC CCC GAC AAG GΨG ΨΨC CGC ΨCC AGC GΨG CΨG CAC ΨCC ACC CAG GAC CΨG ΨΨC CΨG CCG ΨΨC ΨΨC AGC AAC GΨG ACC ΨGG ΨΨC CAC GCC AΨC ΨCC GGC ACC AAC GGC ACC AAG CGG ΨΨC GAC AAC CCC GΨG CΨG CCC ΨΨC AAC GAC GGG GΨG ΨAC ΨΨC GCC AGC ACC GAG AAG ΨCC AAC AΨC AΨC CGC GGC ΨGG AΨC ΨΨC GGC ACC ACC CΨG GAC AGC AAG ACC CAG AGC CΨG CΨG AΨC GΨG AAC AAC GCC ACC AACGΨG GΨC AΨC AAG GΨG ΨGC GAG ΨΨC CAG ΨΨC ΨGC AAC GAC CCC ΨΨC CΨG GAC CAC AAG AAC AAC AAG AGC ΨGG AΨG GAG AGC GGC GΨG ΨAC AGC AGC GCC AAC AAC ΨGC ACC ΨΨC GAG ΨAC GΨG ΨCC CAG CCG ΨΨC CΨG AΨG GAC CΨG GAG GGC AAG CAG GGC AAC ΨΨC AAG AAC CΨG CGC GAG ΨΨC GΨG ΨΨC AAG AAC AΨC GAC GGC ΨAC ΨΨC AAG AΨC ΨAC AGC AAG CAC ACC CCG AΨC AΨC GΨG CGG GAG CCC GAG GAC CΨG CCG CAG GGC ΨΨC ΨCG GCG CΨG GAG CCC CΨG GΨG GAC CΨG CCC AΨC GGC AΨC AAC AΨC ACC CGG ΨΨC CAG ACG CΨG CAC CGC AGC ΨAC CΨG ACC CCC GGC GAC AGC AGC AGC GGG ΨGG ACG GCG GGC GCC GCC GCG ΨAC ΨAC GΨG GGC ΨAC CΨG CAG CCG CGG ACC ΨΨC CΨG CΨG AAG ΨAC AAC GAG AAC GGC ACC AΨC ACC GAC GCC GΨG GAC ΨGC GCG CΨG GAC CCG CΨG AGC GAG ACG AAG ΨGC ACC CΨG AAGGGA GGA GGG GCG GGA GGG GCA GGA GGG GCGCGG GΨG CAG CCC ACC GAG ΨCC AΨC GΨG CGG ΨΨC CCC AAC AΨC ACC AAC CΨG ΨGC CCC ΨΨC GGC GAG GΨG ΨΨC AAC GCC ACC CGC ΨΨC GCC ΨCC GΨG ΨAC GCC ΨGG AAC CGG AAG CGG AΨC AGC AAC ΨGC GΨG GCC GAC ΨAC ΨCC GΨG CΨG ΨAC AAC ΨCC GCC AGC ΨΨC AGC ACC ΨΨC AAG ΨGC ΨAC GGC GΨG ΨCC CCG ACC AAG CΨG AAC GAC CΨG ΨGC ΨΨC ACG AAC GΨG ΨAC GCC GAC AGC ΨΨC GΨG AΨC CGG GGG GAC GAG GΨG CGG CAG AΨC GCC CCC GGC CAG ACG GGC AAC AΨC GCC GAC ΨAC AAC ΨAC AAG CΨG CCC GAC GAC ΨΨC ACC GGC ΨGCGΨG AΨC GCC ΨGG AAC AGC AAC AAC CΨG GAC ΨCC AAG GΨC GGC GGC AAC ΨAC AAC ΨAC CGC ΨAC CGG CΨG ΨΨC CGG AAG ΨCC AAC CΨG AAG CCC ΨΨC GAG CGG GAC AΨC ΨCC ACC GAG AΨC ΨAC CAG GCC GGC AAC AAG CCG ΨGC AAC GGC GΨG AAG GGC ΨΨC AAC ΨGC ΨAC ΨΨC CCG CΨG CAG ΨCC ΨAC GGC ΨΨC CAG CCC ACG ΨAC GGC GΨG GGC ΨAC CAG CCC ΨAC AGG GΨG GΨG GΨG CΨG AGC ΨΨC GAG CΨG CΨG CAC GCC CCG GCC ACC GΨG ΨGC GGC CCG AAG AAG AGC ACC AAC CΨC GΨG AAG AAC AAG ΨGC GΨG AAC ΨΨCΨAAΨΨGΨGΨAΨGCGΨΨAAΨAAAAAGAAGGAACΨCGΨAAAAACΨCAAΨGΨAΨ ΨΨCΨGAGGAAGCGΨGGΨGCAΨAAΨGCCACGCAGCGΨCΨGCAΨAACΨΨΨΨAΨΨAΨ ΨΨCΨΨΨΨAΨΨAAΨCAACAAAAAAAAAAAAAAAAAAAAAAAAAAAAAAAAAAAAAAAA AAAAAAAAAAAAAAAAAAAAAAAAAAAAAAAAAAAAAAAAAAAAAAAAAAAAAAAAA AAAAAAAAAAAAAAAAAAAAAAAAAAAAAAAAAA

Ψ = 1-methyl-3'-pseudouridylyl

(d) Translated Protein of DOW-21

METPAQLLFLLLLWLPDTTGSQCVNLRTRTQLPPAYTNSFTRGVYYPDKVFRSSVLHST
QDLFLPFFSNVTWFHAISGTNGTKRFDNPVLPFNDGVYFASTEKSNIIRGWIFGTTLDSKT
QSLLIVNNATNVVIKVCEFQFCNDPFLDHKNNKSWMESGVYSSANNCTFEYVSQPFLM
DLEGKQGNFKNLREFVFKNIDGYFKIYSKHTPIIVREPEDLPQGFSALEPLVDLPIGINITR
FQTLHRSYLTPGDSSSGWTAGAAAYYVGYLQPRTFLLKYNENGTITDAVDCALDPLSET
KCTLKGGGAGGAGGARVQPTESIVRFPNITNLCPFGEVFNATRFASVYAWNRKRISNCV
ADYSVLYNSASFSTFKCYGVSPTKLNDLCFTNVYADSFVIRGDEVRQIAPGQTGNIADY
NYKLPDDFTGCVIAWNSNNLDSKVGGNYNYRYRLFRKSNLKPFERDISTEIYQAGNKPC
NGVKGFNCYFPLQSYGFQPTYGVGYQPYRVVVLSFELLHAPATVCGPKKSTNLVKNKC
VNF-
