# Supplementary material for: Design and Immunoinformatic Assessment of Candidate Multivariant mRNA Vaccine Construct against Immune Escape Variants of SARS-CoV-2
Source: Polymers (Basel). 2022 Aug 10;14(16):3263. doi: 10.3390/polym14163263 (PMC9414445; doi:10.3390/polym14163263)
Supplement: Supplementary file 1 [file polymers-14-03263-s001.zip › Table S1.pdf]

DOW-21		
**Target Rank**	**miRNA Name**	**Target Score**
**1**	hsa-miR-6868-3p	82
**2**	hsa-miR-3682-3p	79
**3**	hsa-miR-1248	79
**4**	hsa-miR-4635	76
**5**	hsa-miR-4492	75
**6**	hsa-miR-4267	74
**7**	hsa-miR-7161-3p	71
**8**	hsa-miR-4677-5p	69
**9**	hsa-miR-4445-5p	67
**10**	hsa-miR-3912-3p	65
**11**	hsa-miR-4703-3p	62
**12**	hsa-miR-4498	61
**13**	hsa-miR-5009-5p	59
**14**	hsa-miR-653-5p	59
**15**	hsa-miR-4291	57
**16**	hsa-miR-762	56
**17**	hsa-miR-5001-5p	56
**18**	hsa-miR-22-5p	56
**19**	hsa-miR-3085-5p	55
**20**	hsa-miR-7112-5p	54
**21**	hsa-miR-8058	54
**22**	hsa-miR-6742-3p	53
**23**	hsa-miR-3619-5p	51
**24**	hsa-miR-6887-3p	50
